# Exosomal LncRNA–NEAT1 derived from MIF-treated mesenchymal stem cells protected against doxorubicin-induced cardiac senescence through sponging miR-221-3p

**DOI:** 10.1186/s12951-020-00716-0

**Published:** 2020-10-31

**Authors:** Lei Zhuang, Wenzheng Xia, Didi Chen, Yijia Ye, Tingting Hu, Shiting Li, Meng Hou

**Affiliations:** 1 Department of Hepatobiliary Surgery, First Affiliated Hospital, Wenzhou Medical University, Wenzhou, China; 2 Department of Neurosurgery, First Affiliated Hospital, Wenzhou Medical University, Wenzhou, China; 3 Department of Neurosurgery, Xinhua Hospital Affiliated to Shanghai Jiaotong University School of Medicine, Shanghai, 200092 China; 4 Department of Radiation Oncology, First Affiliated Hospital, Wenzhou Medical University, No. 2 Fuxue Lane, Wenzhou, 325000 People’s Republic of China

**Keywords:** Mesenchymal stem cells, Exosome, Macrophage migration inhibitory factor (MIF), Doxorubicin-induced cardiomyopathy, LncRNA-NEAT1/miR-221-3p/Sirt2 signaling pathway

## Abstract

**Background:**

The chemotherapy drug doxorubicin (Dox) is widely used for treating a variety of cancers. However, its high cardiotoxicity hampered its clinical use. Exosomes derived from stem cells showed a therapeutic effect against Dox-induced cardiomyopathy (DIC). Previous studies reported that exosomes derived from mesenchymal stem cells (MSCs) pretreated with macrophage migration inhibitory factor (MIF) (exosome^MIF^) showed a cardioprotective effect through modulating long noncoding RNAs/microRNAs (lncRNAs/miRs). This study aimed to investigate the role of exosome^MIF^ in the treatment of DIC.

**Results:**

Exosomes were isolated from control MSCs (exosome) and MIF-pretreated MSCs (exosome^MIF^). Regulatory lncRNAs activated by MIF pretreatment were explored using genomics approaches. Fluorescence-labeled exosomes were tracked in vitro by fluorescence imaging. In vivo and in vitro, miR-221-3p mimic transfection enforced miR-221-3p overexpression, and senescence-associated β-galactosidase assay was applied to test cellular senescence. Exosomal delivering LncRNA-NEAT1 induced therapeutic effect in vivo was confirmed by echocardiography. It demonstrated that exosomes^MIF^ recovered the cardiac function and exerted the anti-senescent effect through LncRNA–NEAT1 transfer against Dox. TargetScan and luciferase assay showed that miR-221-3p targeted the Sirt2 3′-untranslated region. Silencing LncRNA–NEAT1 in MSCs, miR-221-3p overexpression or Sirt2 silencing in cardiomyocytes decreased the exosome^MIF^-induced anti-senescent effect against Dox.

**Conclusions:**

The results indicated exosome^MIF^ serving as a promising anti-senescent effector against Dox-induced cardiotoxicity through LncRNA–NEAT1 transfer, thus inhibiting miR-221-3p and leading to Sirt2 activation. The study proposed that exosome^MIF^ might have the potential to serve as a cardioprotective therapeutic agent during cancer chemotherapy.
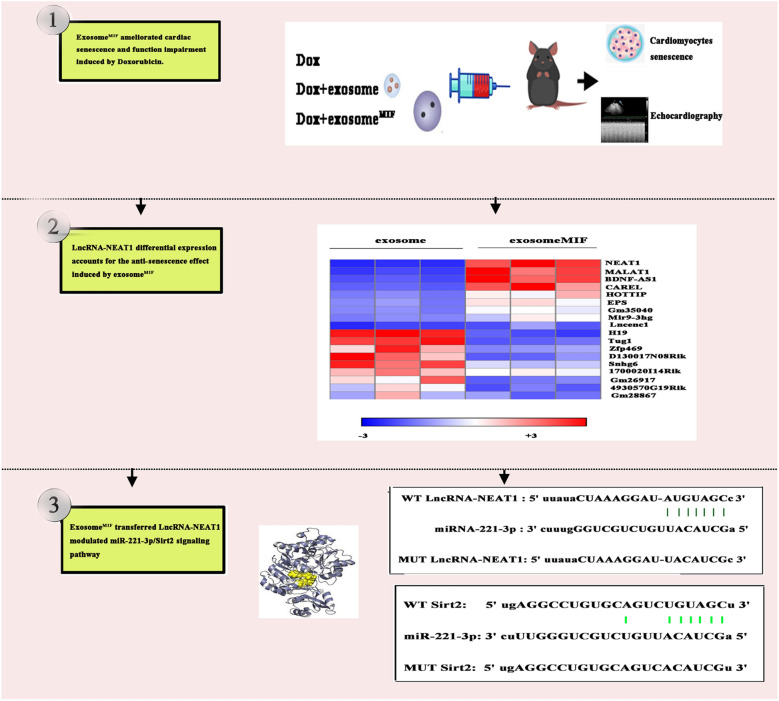

## Background

Advances in cancer chemotherapy have led to a remarkable decrease in mortality rates and, consequently, a rapid increase in the number of cancer survivors [1]. Many of these cancer survivors suffer from chemotherapy-related cardiac complications later in life. Cardiovascular adverse effects have become the second leading cause of death in these patients, following recurrent malignancy [2]. The anthracycline antibiotic doxorubicin (Dox) is a very effective anti-cancer agent. However, up to 28 percent of treated patients occurred cardiovascular complications [3, 4]. The major mechanism by which Dox induces cardiac damages involves disruption of DNA and RNA synthesis [5], oxidative damage via the formation of reactive oxygen species (ROS) [6], changes in mitochondrial membrane potential ΔΨm [7], all above were the main reasons for cellular senescence [8]. Therefore, further exploration of the method alleviating Dox-induced cardiac senescence is warranted for the development of effective cardioprotective strategies.

Bone marrow-derived mesenchymal stem cells (BM-MSCs) are an emerging and promising approach to treat cardiac injuries [9, 10]. However, limitations and challenges cannot be ignored when transplanting MSCs directly into target tissues. Studies have reported that the survival rate of transplanted stem cells is very low, hampering the transplantation efficiency and hence leading to impaired treatment effect [11, 12]. There is mounting evidence that MSCs related repairment or regeneration of damaged cardiac tissues, primarily by means of exosomes release, which are safer and more effective [13]. At meanwhile, the contribution of exosomes to could be further improved though modifying MSCs [14]. When developing exosome-based therapeutics, exosomes were found to elicit differential effects on recipient cells depending on the cultural environment of parent cells [15]. These findings led to the search for a culture condition to optimize exosomes to be more efficient in treating Dox-induced cardiac injury. Migration inhibitory factor (MIF) is involved in multiple cardiac injuries, including cardiac senescence [16, 17]. In older individuals, tolerance to ischemic stress is reduced, accompanied by impaired MIF expression. A previous study showed that regaining MIF function attenuated cardiac injury [18]. Beneficial cardioprotective effects of exosomes derived from MSCs have also been reported in the pretreatment with MIF [19]. Given the potential therapeutic benefits of exosomes derived from MSCs pretreated with MIF (exosome^MIF^), whether MIF pretreatment could affect the functions of exosomes in treating Dox-related cardiac senescence process needs to be determined.

Intriguingly, the existence of long noncoding RNAs (lncRNAs) in exosomes has been reported, suggesting that lncRNAs may also be loaded with exosomes to regulate gene expression in host cells via cell-to-cell communication [20]. LncRNA–NEAT1 is located on chromosome 11 and has been indicated to play a significant role in the cardioprotective process through MSC-derived exosome transfer [19]. Besides, the participation of lncRNA–NEAT1 has been elucidated to be beneficial in the inhibition of Dox-induced cardiotoxicity and apoptosis [21]. Current insights into the molecular systems of lncRNA–miRNA regulatory interactions and implications in the treatment of Dox-induced cardiotoxicity allow a hypothesis that lncRNA–NEAT1 may be involved in the regulatory network of mRNA via competing endogenous RNAs (ceRNAs)-mediated miRNA evasion [22]. A target ceRNA of lncRNA–NEAT1, miR-221-3p, impaired tissue regeneration through inhibiting the sirtuin (Sirt) family of proteins [23]. A previous study reported that exosome-transferred LncRNA–NEAT1 activated the Sirt protein family to exert a cardioprotective effect [19].

Therefore, the present study was performed to investigate the potential therapeutic effect of the exosome^MIF^-transferred LncRNA–NEAT1, inhibited miR-221-3p, in Dox-induced cardiac senescence to provide a feasible therapeutic target for Dox-induced cardiotoxicity.

## Results

### Exosome^MIF^ alleviated Dox-induced cardiac injury

**Fig. 1:**
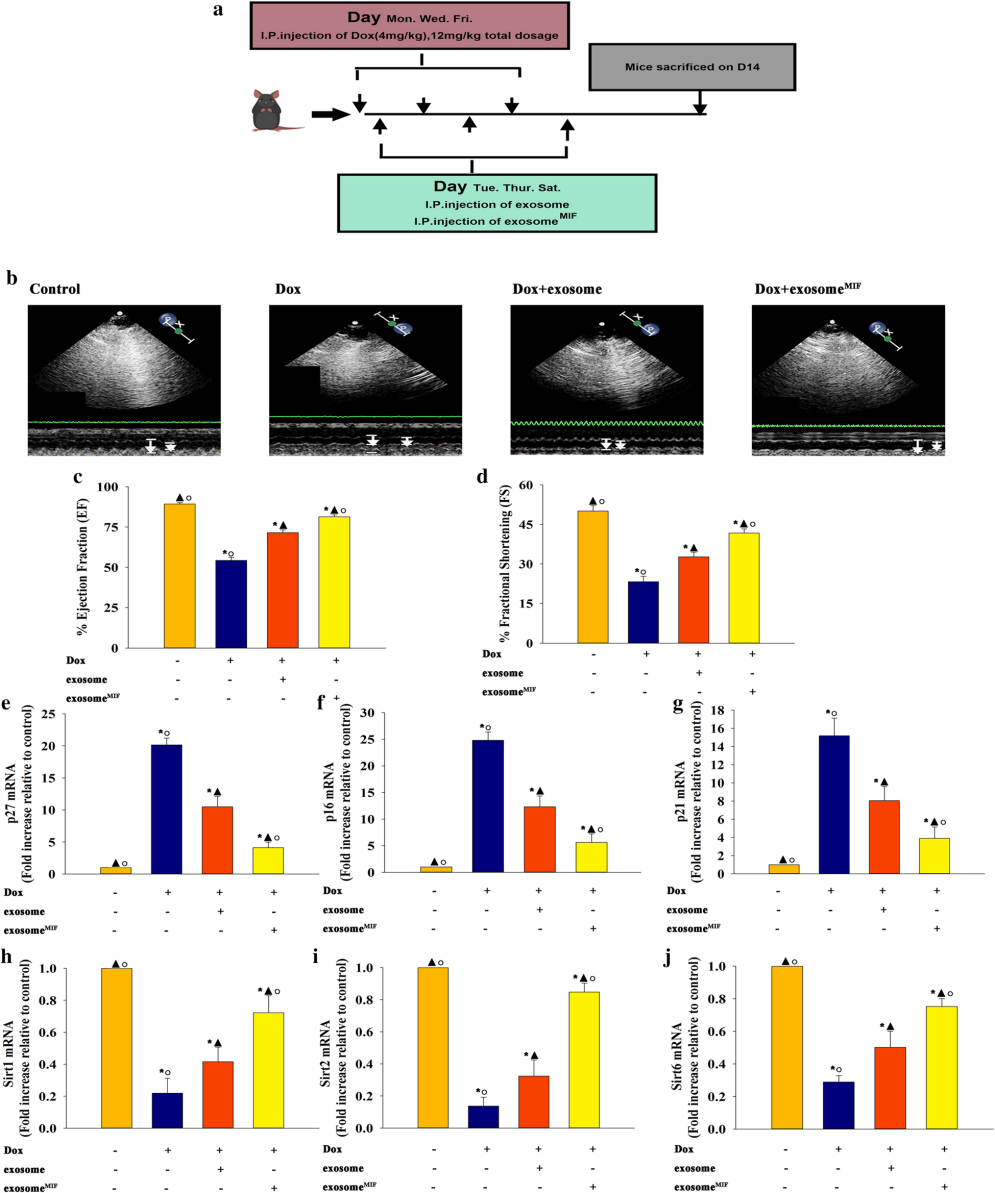
Exosomes derived from MSCs pretreated with MIF alleviated Dox-induced cardiac injury. **a** The schema of the mice treatment. **b** Representative images of echocardiography exhibiting changes in cardiac function in each group. Echocardiographic analysis of ejection fraction (EF) **c** and fractional shortening (FS) **d** in week 2 after the first cycle of Dox, Dox + exosome, Dox + exosome^MIF^, or control treatment. **e**–**j** The expression of senescence-related genes *p27* (E), *p16* (**f**), and *p21* (**g**), and rejuvenation-related genes *Sirt1* (**h**), *Sirt2* (**i**), and *Sirt6* (**j**) in the aforementioned groups was examined using qRT-PCR. Each column represents the mean ± SD of three independent experiments. *P < 0.05 versus Control; ^▲^*P* < 0.05 versus Dox; ^○^*P* < 0.05 versus Dox + exosome in one-way analysis of variance, n = 3

Eight-week-old mice received Dox treatment to simulate clinical chemotherapy (Fig. 1a). Echocardiography showed that left ventricular ejection fraction (EF) and fractional shortening (FS) significantly decreased, which was recovered by exosomes^MIF^, in the Dox group compared with the control group. In contrast, the values in the exosome group indicated a therapeutic effect; however, it was not significant compared with the exosome^MIF^ group (Fig. 1b–d). The expression of senescence-related and rejuvenation-related genes in mouse hearts were compared to confirm cellular senescence modulated by exosomes^MIF^ at the organ level. The expression levels of senescence-related genes *p27*, *p16*, and *p21* significantly increased (Fig. 1e–g), while the expression levels of rejuvenation-related genes *Sirt1*, *Sirt2*, and *Sirt6* decreased (Fig. 1h–j) in the Dox group compared with the Dox + exosome^MIF^ group.

### Exosome^MIF^ prevented the cardiac injury process mediated by LncRNA–NEAT1 transfer

**Fig. 2:**
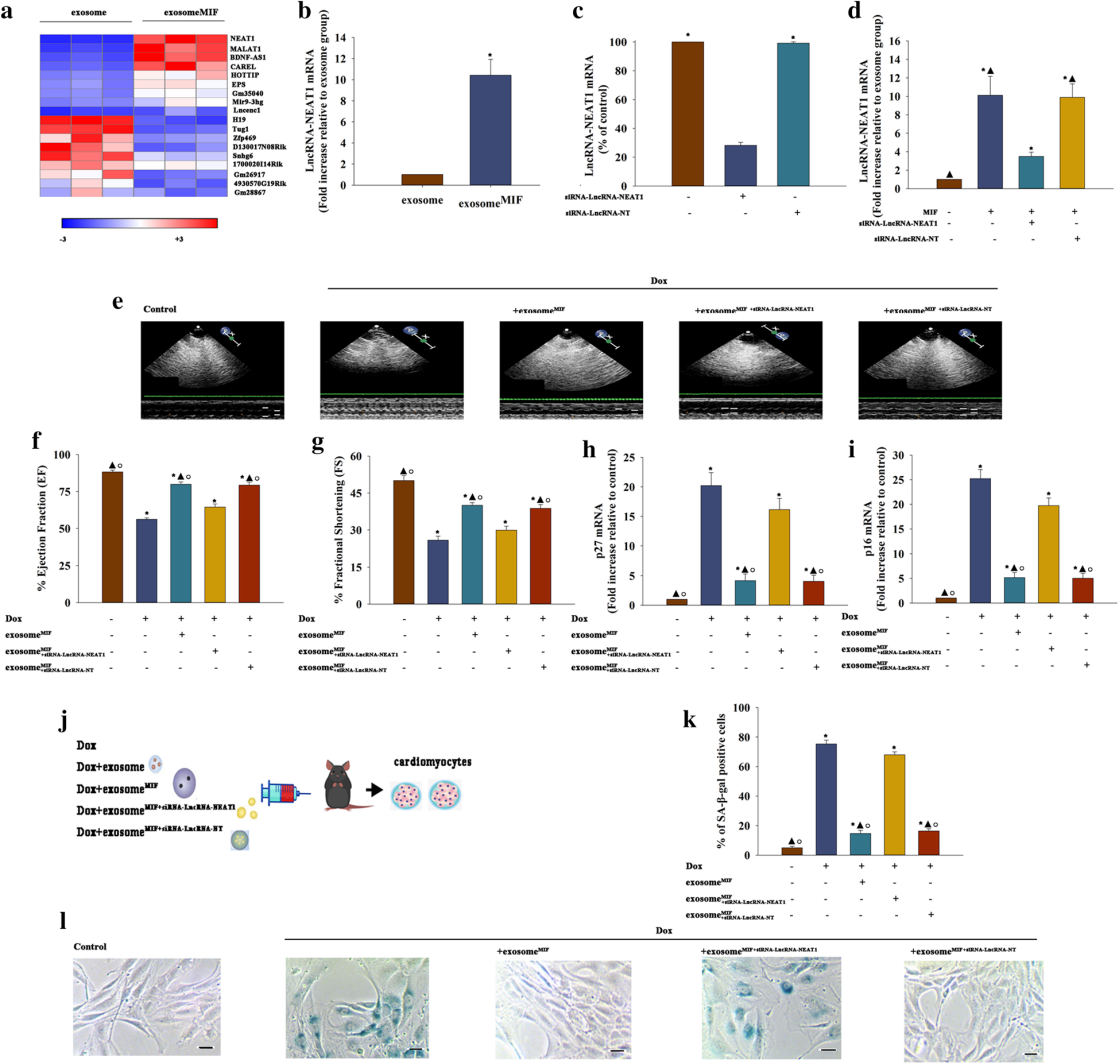
Exosome^MIF^ prevented the cardiac injury process mediated by LncRNA–NEAT1 transfer. **a** Heat map of lncRNAs differentially expressed between exosome and exosome^MIF^. **b** LncRNA–NEAT1 expression was validated by qRT-PCR in exosome and exosome^MIF^. *P < 0.05 vs exosome in Student’s t-test, n = 3. **c** LncRNA–NEAT1 expression was validated by qRT-PCR in MSCs after LncRNA–NEAT1 silencing. *P < 0.05 vs siRNA–LncRNA–NEAT1 in repeated measures ANOVA, n = 3. **d** LncRNA–NEAT1 expression was validated by qRT-PCR in exosomes after silencing LncRNA–NEAT1 in MSCs. *P < 0.05 vs Control; ^▲^*P* < 0.05 vs MIF + siRNA−LncRNA−NEAT1 in repeated measures ANOVA, n = 3. **e** Representative images of echocardiography exhibiting the changes in cardiac function in each group. Echocardiographic analysis of EF (**f**) and FS (**g**). **h**, **i** *p*27 and *p*16 mRNA levels were analyzed using qRT-PCR. **j** Cardiomyocytes were isolated from control, Dox−, Dox + exosome^MIF^−, Dox + exosome^MIF+siRNA−LncRNA−NEAT1^-, and Dox + exosome^MIF+siRNA−LncRNA−NT^-treated mice. **k** Percentage of β-gal-positive cells. **l** Representative images of SA-β-gal staining. Each column represents mean ± SD of three independent experiments. *P < 0.05 versus Control; ^▲^*P* < 0.05 versus Dox; ^○^*P* < 0.05 versus Dox+ exosome^MIF+siRNA−LncRNA−NEAT1^ in one-way analysis of variance, n = 3

The microarray analysis between exosome and exosome^MIF^ was performed to determine whether exosomes could prevent the senescence process mediated by lncRNA. As shown in Fig. 2a, LncRNA–NEAT1 stood out as a candidate probably responsible for the anti-senescence effect. QRT-PCR further confirmed that LncRNA–NEAT1 was more abundant in exosome^MIF^ (Fig. 2b). The siRNA targeting LncRNA–NEAT1 could downregulate the expression of LncRNA–NEAT1 in MSCs and exosomes (Fig. 2c, d). Exosome^MIF^ attenuated the effects of Dox on cardiac function, and siRNA–LncRNA–NEAT1 blocked the function of exosome^MIF^ (Fig. 2e–g). It also attenuated the inducement of Dox on senescent-related genes *p27* and *p*16; the beneficial effects of exosomes were blocked by siRNA–LncRNA–NEAT1 (Fig. 2j, i). Cardiomyocytes were isolated from control and Dox-, Dox + exosome^MIF^-, Dox + exosome^MIF+siRNA−LncRNA−NEAT1^-, and Dox + exosome^MIF+siRNA−LncRNA−NT^-treated mice (Fig. 2j). SA-β-gal staining revealed more positive cells in Dox-treated than in control mouse hearts, while exosome^MIF^ treatment recovered senescence. Moreover, silencing LncRNA–NEAT1 decreased the effect of exosome^MIF^ (Fig. 2k, l).

### Exosome^MIF^ treatment reduced Dox-related cardiac injury through inhibiting miR-221-3p

**Fig. 3:**
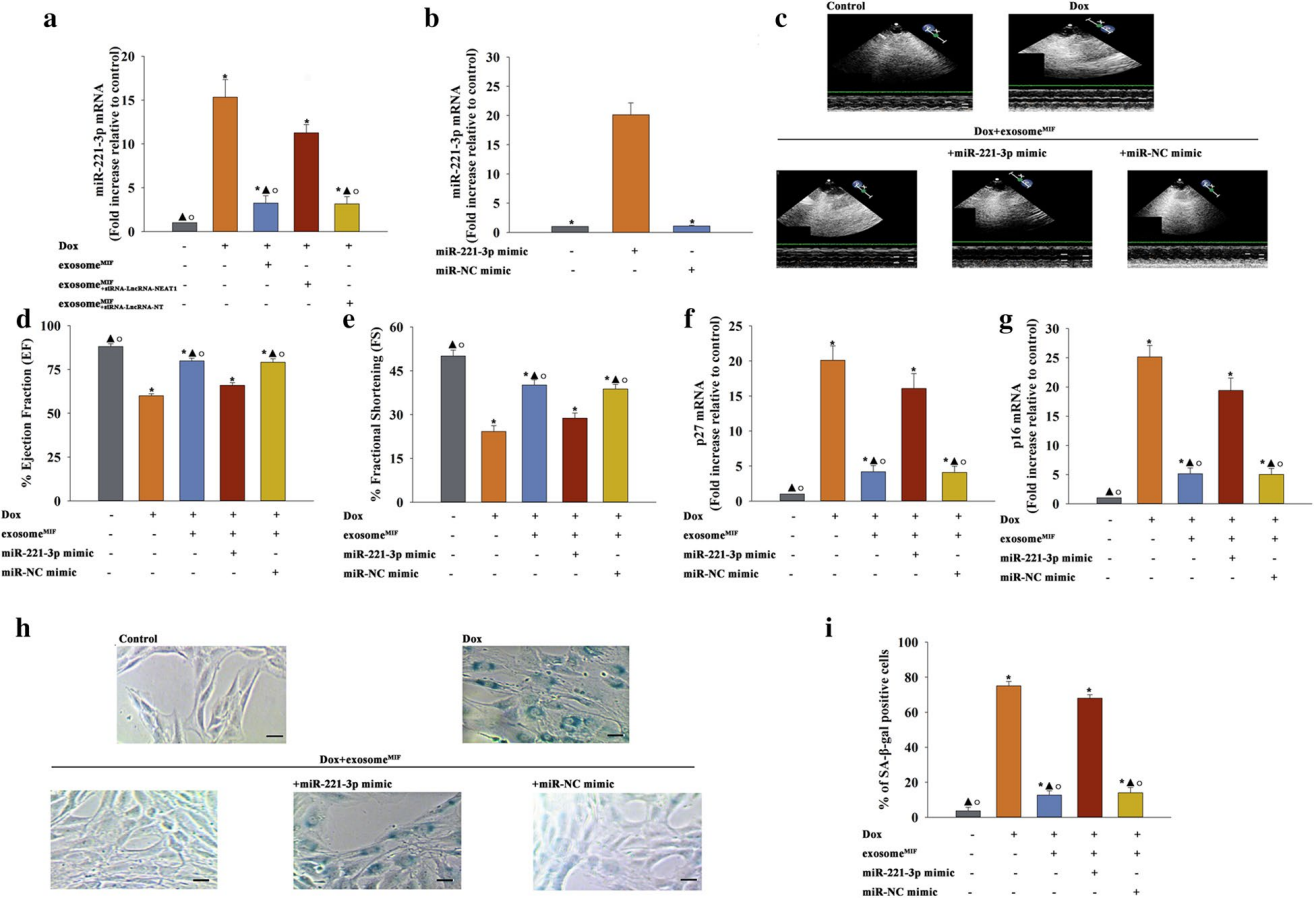
Exosome^MIF^ treatment reduced Dox-related cardiac injury through inhibiting miR-221-3p. **a** MiR-221-3p expression was validated by qRT-PCR in cardiac tissue. *P < 0.05 versus Control; ^▲^*P* < 0.05 versus Dox; ^○^*P* < 0.05 versus Dox + exosome^MIF+siRNA−LncRNA−NEAT1^ in one-way analysis of variance, n = 3. **b** Overexpression effect was validated by qRT-PCR. *P < 0.05 versus miR-221-3p mimic in one-way analysis of variance, n = 3. **c** Representative images of echocardiography exhibiting changes in cardiac function in each group. Echocardiographic analysis of EF (**d**) and FS (**e**) in week 2 after treatment. **f**, **g** *p*27 and *p*16 mRNA levels were analyzed using qRT-PCR. **h** Representative images of SA-β-gal staining. **i** Percentage of β-gal-positive cells. Each column represents the mean ± SD of three independent experiments. *P < 0.05 vs Control; ^▲^*P* < 0.05 versus Dox; ^○^*P* < 0.05 versus Dox + exosome^MIF^ + miR-221-3p mimic in one-way analysis of variance, n = 3

Recent studies suggested the upregulation of miR-221-3p during the cellular senescence process [24]. The upregulation of this miR was also shown in Dox-treated hearts. However, this significant increase in miR-221-3p was reduced following exosome^MIF^ treatment and blocked by siRNA–LncRNA–NEAT1 in MSCs, even in those treated with MIF (Fig. 3a). MiR-221-3p mimic transfection was performed in vivo to evaluate whether exosome^MIF^-induced miR-221-3p inhibition influenced cardiac senescence and cardiac function impaired by Dox (Fig. 3b). Echocardiographic data revealed that Dox-treated mice had significantly impaired cardiac function compared with control mice. However, exosome^MIF^ showed a therapeutic effect, which was suppressed by miR-221-3p overexpression (Fig. 3c–e). Meanwhile, the expression levels of *p*27 and *p*16 mRNAs also decreased on treatment with exosome^MIF^ (Fig. 3f, g). SA-β-gal staining results revealed that the number of senescent cardiomyocytes decreased in the exosome^MIF^-treated group (Fig. 3h, i). These anti-senescent effects of exosome^MIF^ were blocked by miR-221-3p mimic transfection.

### Exosome^MIF^ had a better anti-senescent effect in cardiomyocytes treated with Dox

**Fig. 4:**
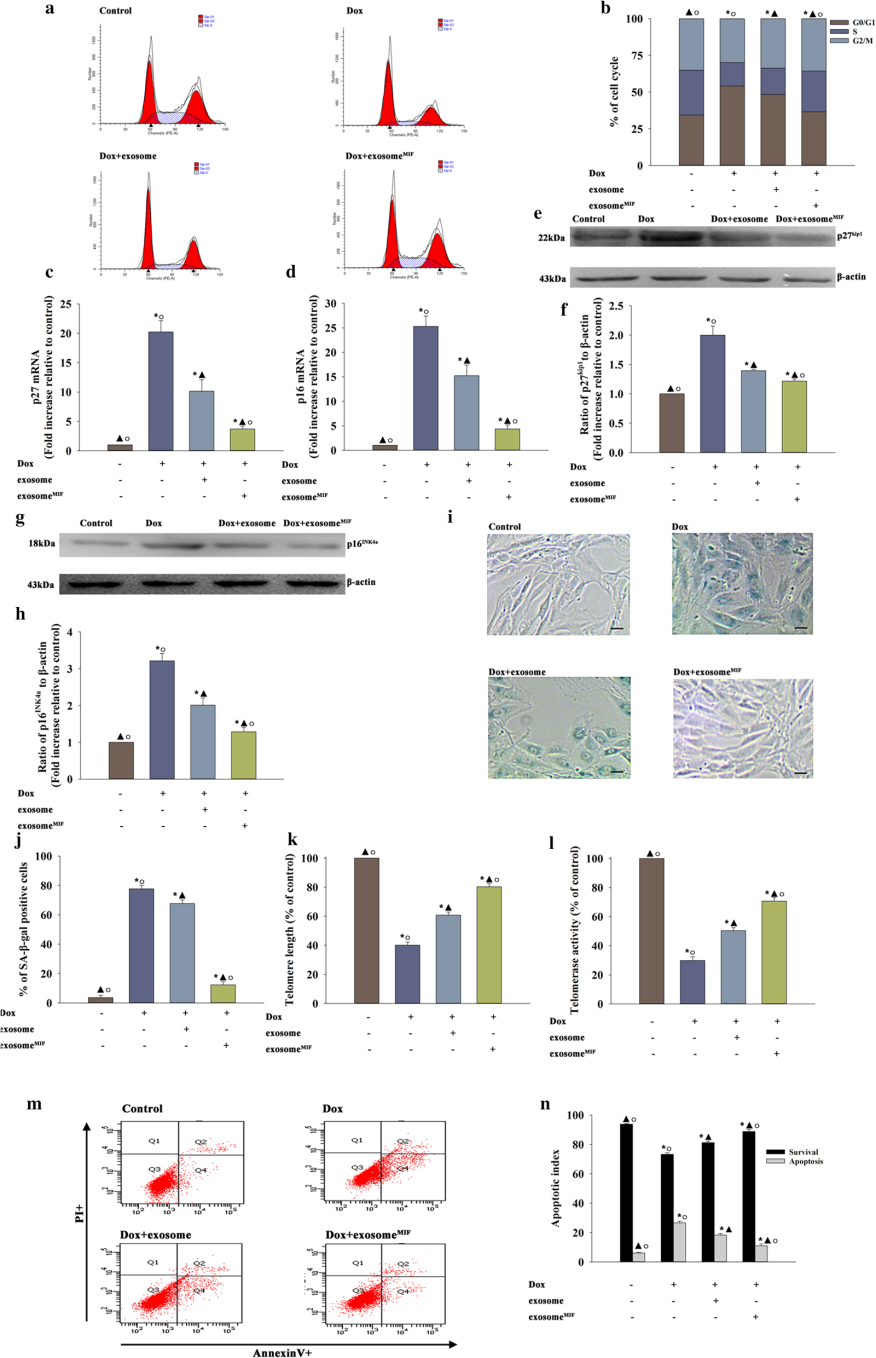
Exosome^MIF^ had a better anti-senescent effect in cardiomyocytes treated with Dox. **a**, **b** Cell cycle distribution was analyzed. **c**, **d** *p*27 and *p*16 mRNA levels were analyzed using qRT-PCR. **e**–**h** p27 and p16 protein levels were analyzed using western blot analysis. **i** Representative images of SA-β-gal staining. **j** Percentage of β-gal-positive cells. **k** The Telomere length was analyzed by qRT-PCR. **l** Relative telomerase activity was measured. **m**, **n** Representative flow cytometric dot plots of apoptotic cells after Annexin V/propidium iodide staining. *P < 0.05 versus Control; ^▲^*P* < 0.05 versus Dox; ^○^*P* < 0.05 versus Dox + exosome in repeated measures ANOVA, n = 3

Exosomes isolated from MSCs showed a round morphology and size of 50–100 nm, according to transmission electron microscope (TEM) (Additional file 1: Fig. S1A) and nanoparticle tracking analysis (NTA) (Additional file 1: Fig. S1B). At meanwhile, the exosome markers Flotillin-1 and CD81 were confirmed by western blot (Additional file 1: Fig. S1C). Considering the anti-senescent effect of MIF, whether MIF pretreatment showed a more cardioprotective effect in exosomes needed to be explored. The effects of exosome^MIF^ on Dox-induced cardiomyocyte senescence were determined. The results showed that exosome^MIF^ protected cardiomyocytes from senescence compared with Dox-treated cardiomyocytes, showing that more cells escaped from the G0/G1 phase as measured using flow cytometric analysis cell scan (FACS) (Fig. 4a, b), with the decreased expression of the cellular senescence–related genes *p27* and *p16* (Fig. 4c–h), a lower percentage of SA-β-gal-positive cells (Fig. 4i, j), but an increase in telomere length and activity (Fig. 4k, l). However, exosomes from MSCs without any treatment also elicited cellular rejuvenation, however not as significant as with exosome^MIF^, indicating that pretreatment with MIF showed a better anti-senescent effect. At meanwhile, 48 h after Dox treatment, cellular apoptosis happened, exosome and exosome^MIF^ both showed protective effect against apoptosis. However, exosomes from untreated MSCs showed less cellular protection, indicating that the observed effects were MIF-specific (Fig. 4m, n).

### Exosome^MIF^ inhibited cardiomyocyte senescence by delivering LncRNA–NEAT1

**Fig. 5:**
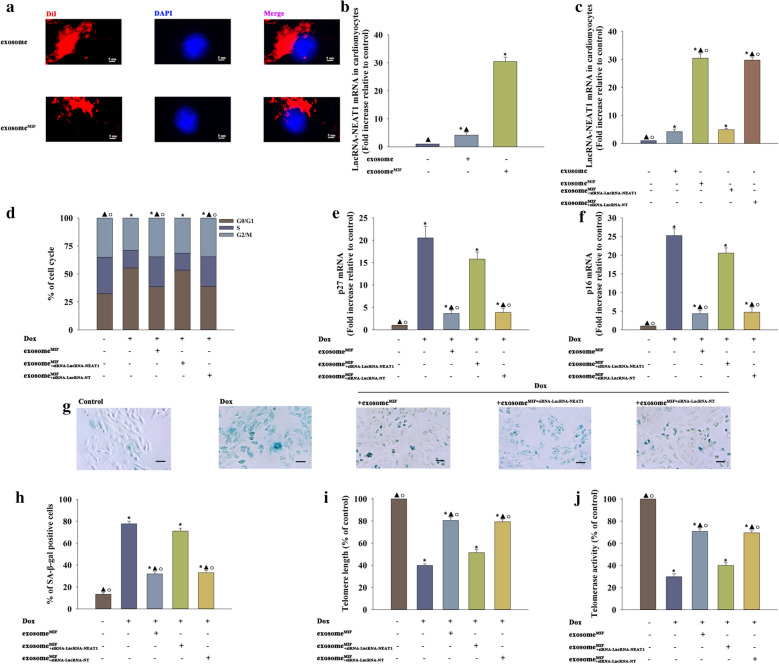
Exosome^MIF^ inhibited cardiomyocyte senescence by delivering LncRNA–NEAT1. **a** DiI-labeled exosome transfer is shown. **b** LncRNA–NEAT1 mRNA in cardiomyocytes was detected using qRT-PCR. *P < 0.05 vs control; ^▲^*P* < 0.05 vs exosome^MIF^ in one-way analysis of variance, n = 3. SiRNA was used to silence LncRNA–NEAT1 in MSCs. **c** QRT-PCR was applied to test LncRNA–NEAT1 mRNA in cardiomyocytes after LncRNA–NEAT1 silencing in MSCs. *P < 0.05 versus Control; ^▲^*P* < 0.05 versus exosome;^○^*P* < 0.05 versus exosome^MIF+siRNA−LncRNA−NEAT1^ in repeated measures ANOVA, n = 3. **d** Cell cycle distribution was analyzed. **e**, **f** *p*27 and *p*16 mRNA levels were analyzed using qRT-PCR. **g** Representative images of SA-β-gal staining. **h** Percentage of β-gal-positive cells. **i** Telomere length was analyzed by qRT-PCR. **j** Relative telomerase activity was measured. *P < 0.05 versus Control; ^▲^*P* < 0.05 versus Dox; ^○^*P* < 0.05 versus Dox + exosome^MIF+siRNA−LncRNA−NEAT1^ in repeated measures ANOVA, n = 3

Notably, the increase in exosome^MIF^-induced LncRNA–NEAT1 showed a significant therapeutic effect against Dox-related cardiac injury. Therefore, the subsequent mechanistic studies focused on LncRNA–NEAT1 in vitro. Exosomes prepared from MSCs were labeled with DiI to confirm whether this lncRNA could be transferred to cardiomyocytes through exosomes. Exosome uptake by cardiomyocytes was observed (Fig. 5a). Then, LncRNA–NEAT1 mRNA was detected in cardiomyocytes incubated with exosomes or exosome^MIF^ compared with cardiomyocytes without any treatment; the untreated cardiomyocytes were used as control. As expected, LncRNA–NEAT1 had the strongest upregulation in cardiomyocytes incubated with exosome^MIF^ (Fig. 5b). An siRNA–LncRNA–NEAT1 was constructed for silencing this lncRNA to further identify the anti-senescent effect of LncRNA–NEAT1 transferred by exosome^MIF^. MSCs were transfected with siRNA against LncRNA–NEAT1 or control siRNA–NT and subjected to MIF. Then, exosomes were collected. Exosomes derived from siRNA against LncRNA–NEAT1 or control siRNA–NT in MSCs transfected and treated with MIF, or MSCs treated with only MIF, were added to cardiomyocytes. In parallel experiments, cardiomyocytes without any treatment were used as the control. QRT-PCR results suggested that exosome^MIF^ transferred LncRNA–NEAT1 to cardiomyocytes, while silencing LncRNA–NEAT1 in MSCs blocked the transfer process (Fig. 5c).

In the subsequent experiments, exosome^MIF^ significantly reduced the percentage of cells in the G0/G1 phase (Fig. 5d), expression of *p*27 and *p*16 (Fig. 5e, f), and number of SA-β-gal-positive cells (Fig. 5g, h). However, it elevated the telomere length and activity (Fig. 5i, j). This protective effect against Dox was inhibited by silencing LncRNA–NEAT1 in MSCs before treatment with MIF. These results supported that exosome^MIF^ exerted an anti-senescent effect through direct LncRNA–NEAT1 transfer.

### LncRNA–NEAT1 transferred by exosome^MIF^ sponged miR-221-3p against cardiomyocyte senescence

**Fig. 6:**
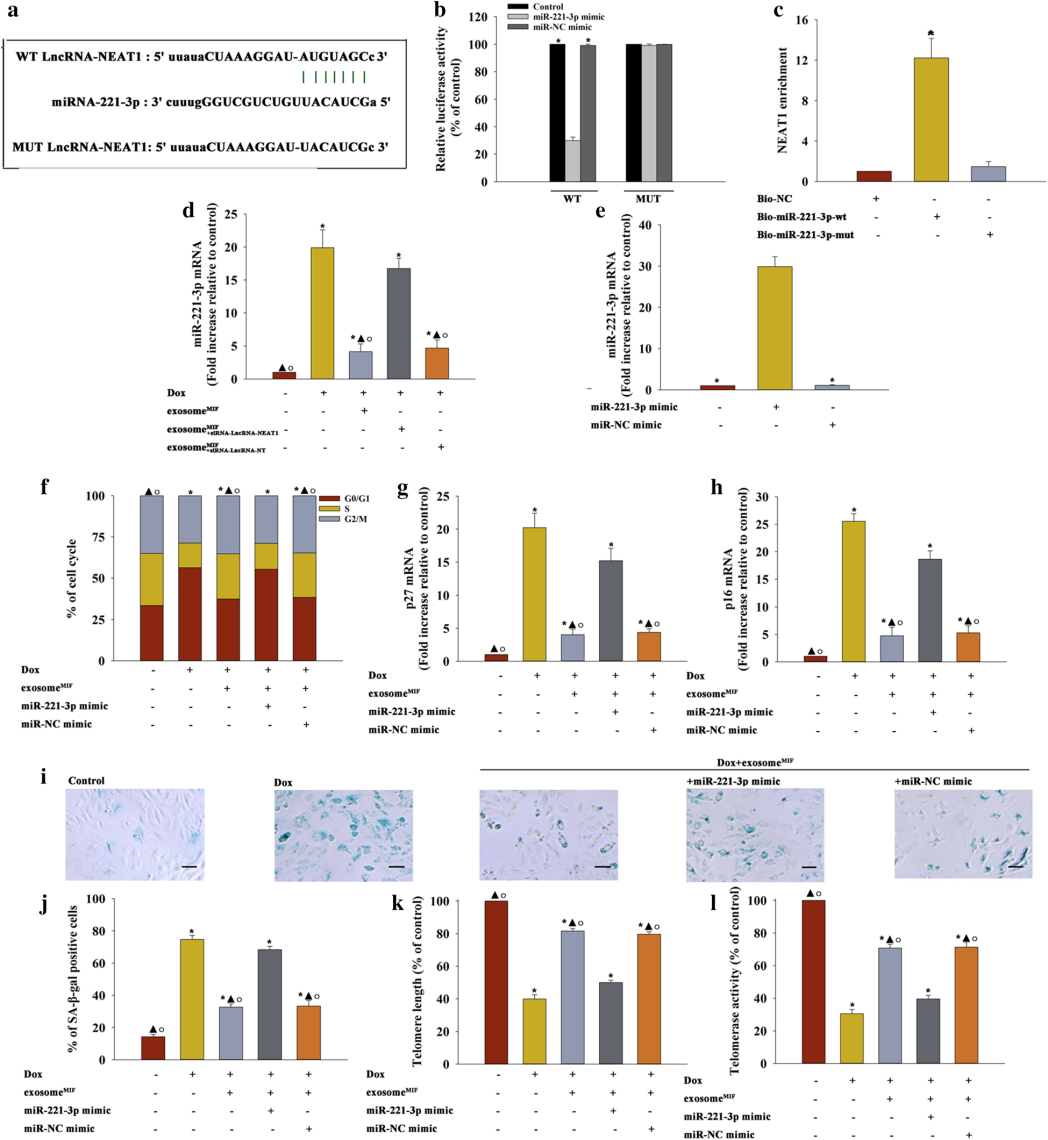
LncRNA–NEAT1 transferred by exosome^MIF^ sponged miR-221-3p against cardiomyocyte senescence. **a** Binding sites of LncRNA–NEAT1 and miR-221-3p. **b** Dual-luciferase reporter. *P < 0.05 versus miR-221-3p mimic in the WT group in repeated measures ANOVA, n = 3. **c** NEAT1 is associated with miR-221-3p, confirmed by biotin-based pulldown assay; *P < 0.05 versus Bio-NC in repeated measures ANOVA, n = 3. **d** miR-221-3p mRNA levels were analyzed by qRT-PCR. *P < 0.05 versus Control; ^▲^*P* < 0.05 versus Dox; ^○^*P* < 0.05 versus Dox+ exosome^MIF+siRNA−LncRNA−NEAT1^ in repeated measures ANOVA, n = 3. **e** Overexpression effect was validated by qRT-PCR. *P < 0.05 versus miR-221-3p mimic in repeated measures ANOVA, n = 3. **f** Cell cycle distribution was analyzed. **g**, **h** *p*27 and *p*16 mRNA levels were analyzed using qRT-PCR. **i** Representative images of SA-β-gal staining. **j** Percentage of β-gal-positive cells. **k** Telomere length was analyzed by qRT-PCR. **l** Relative telomerase activity was measured. *P < 0.05 vs Control; ^▲^*P* < 0.05 vs Dox; ^○^*P* < 0.05 vs Dox+ exosome^MIF^ + miR-221-3p mimic in repeated measures ANOVA, n = 3

Furthermore, the miRcode database was applied to predict the potential target miRNAs with a sequence complementary to that of LncRNA–NEAT1. LncRNA–NEAT1 contains the binding site for miR-221-3p, suggesting that lncRNA as a ceRNA sponged miR-221-3p to limit its function (Fig. 6a), which was confirmed by dual-luciferase gene reporter assay. As illustrated in Fig. 6b, the co-transfection of miR-221-3p significantly inhibited the luciferase activities elicited by LncRNA–NEAT1. Further, a biotin–avidin pulldown system was employed to test whether miR-221-3p could pull down NEAT1. Cardiomyocytes were transfected with biotinylated miR-221-3p, then collected for biotin based pulldown assay. NEAT1 was pulled down as analyzed by qRT-PCR (Fig. 6c). To confirm whether exosomal derived LncRNA-NEAT1 acted as a ceRNA to sponge miR-221-3p, qRT-PCR was applied to quantify the miR-221-3p expression. MiR-221-3p was induced by Dox in cardiomyocytes but substantially reduced by exosome^MIF^. However, this repressive effect was impaired by exosome^MIF^ derived from MSCs with silenced LncRNA–NEAT1 (Fig. 6d). Importantly, miR-221-3p overexpression (Fig. 6e) markedly increased the number of cells in the G0/G1 phase (Fig. 6f), expression of *p27* and *p16* (Fig. 6g, h), and number of SA-β-gal-positive cells (Fig. 6i, j), but inhibited the telomere and telomerase activity (Fig. 6k, l), even when exosome^MIF^ were added to Dox-treated cardiomyocytes (Fig. 6f–l).

### MiR-221-3p shuttling by exosome^MIF^ reversed senescence through targeting Sirt2

**Fig. 7:**
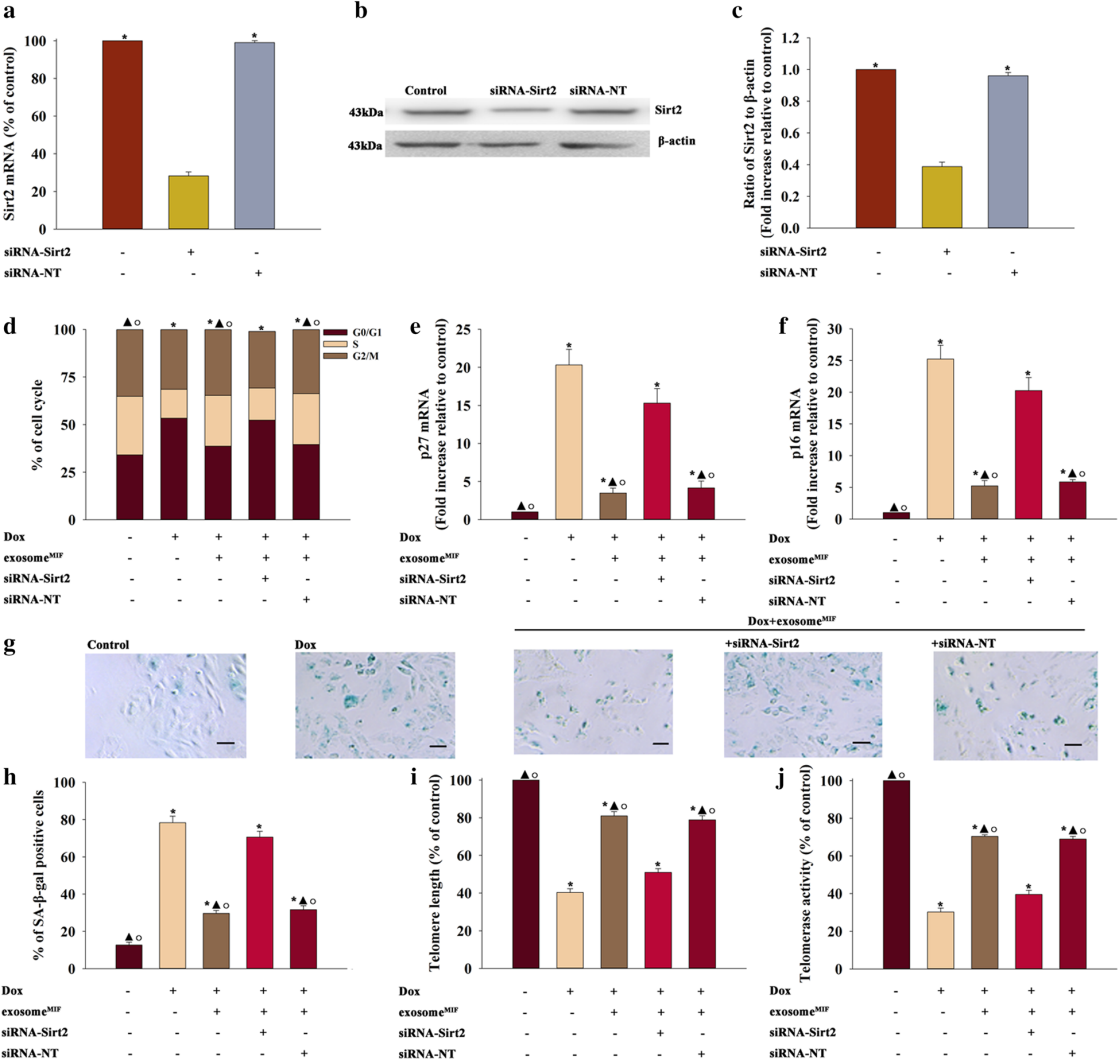
MiR-221-3p shuttling by exosome^MIF^ reversed senescence through targeting Sirt2. **a**–**c** QRT-PCR (**a**) and western blot analysis (**b**, **c**) tested the siRNA-mediated transfection efficiency. Each column represents the mean ± SD from three independent experiments. *P < 0.05 versus siRNA-Sirt2 in repeated measures ANOVA, n = 3. **d** Cell cycle distribution was analyzed. **e**, **f** *p*27 and *p*16 mRNA levels were analyzed using qRT-PCR. **g** Representative images of SA-β-gal staining. **h** Percentage of β-gal-positive cells. **i** Telomere length was analyzed by qRT-PCR. **j** Relative telomerase activity was measured. *P < 0.05 versus Control; ^▲^*P* < 0.05 versus Dox; ^○^*P* < 0.05 versus Dox + exosome^MIF^ + siRNA-Sirt2 in repeated measures ANOVA, n = 3

To better understand how exosome^MIF^ modulated exosome^MIF^-induced cardiac senescence, the study focused on the target gene of miR-221-3p within cardiomyocytes. The database of miRNA targets indicated that Sirt2 might be the potential target relative to the anti-senescent effect. As shown in Additional file 2: Figure S2A, the 3′-UTR of Sirt2 contains a binding site for miR-221-3p. The dual-luciferase gene reporter assay revealed that the relative luciferase activity was significantly weakened in the Sirt2-WT + miR-221-3p mimic group (Additional file 2: Fig. S2B). As expected, Dox treatment markedly inhibited Sirt2 expression in cardiomyocytes, attenuated by exosome^MIF^. In addition, elevating miR-221-3p expression impaired the expression of Sirt2 (Additional file 2: Fig. S2C and 2D). These results suggested that miR-221-3p inhibited the expression of Sirt2 via post-transcriptional regulation. This study further investigated the mechanism underlying the crosstalk between Sirt2 and exosome^MIF^ in Dox-induced cellular senescence by silencing Sirt2 using siRNA. Sirt2 mRNA and protein expression levels were significantly reduced in cells transfected with siRNA-Sirt2 compared with cells transfected with nontargeting siRNA (siRNA–NT) as the control (Fig. 7a–c). Sirt2 silencing markedly increased the number of cells in the G0/G1 phase (Fig. 7d), expression of *p27* and *p16* (Fig. 7e, f), and number of SA-β-gal-positive cells (Fig. 7g, h), but impaired the telomere length and telomerase activity (7I and J), even when exosome^MIF^ were added to Dox-treated cardiomyocytes.

## Discussion

Dox is one of the most potent broad-spectrum antitumor anthracycline antibiotics commonly used to treat a variety of cancers [25]. However, the clinical use of Dox is limited because of its serious cardiotoxicity, which often leads to irreversible degenerative cardiomyopathy and heart failure [26]. The most common complications of Dox cardiotoxicity are left ventricular dysfunction [3], coincidence with the findings of this study showing that Dox treatment impaired left ventricular function, accompanied by lower EF and FS values. The mechanism of Dox-induced cardiotoxicity involves the disruption of DNA and RNA synthesis, eventually leading to cell senescence [27]. In the present study, the cellular senescence-dependent increase in Dox-induced cardiac dysfunction was associated with increased cell cycle arrest, which is the main phenotype of the disruption of DNA and RNA synthesis and senescence of cardiomyocytes [28].

Substantial evidence is available showing that stem cells exert their therapeutic effect via the secretion of soluble factors and the production of exosomes [29]. The content in exosomes may vary depending on the physiological and pathological states of culture conditions [30]. This study showed that MIF activation contributed to modifying exosomes^MIF^ derived from MSCs to exert the anti-senescent effect after Dox-induced cardiac injury. In addition, exosomes^MIF^ recovered the telomere length and activity, which were important in heart regeneration [31]. Exosomes alone showed a therapeutic effect to some extent [32]. However, it is conceivable that exosomes related to better anti-senescent effects might be mediated by treatment with anti-senescent and cardioprotective factor MIF.

A number of recent studies have revealed that lncRNAs play an important role in senescence-related cardiac injury [33]. Some lncRNAs contained in exosomes serve as important messages to alter gene expression and cellular functions of distant organs, usually acting as regulators in many cardiac pathological processes [14]. Modifying a cell culture environment, leading to changes in components of lncRNAs, and investigating the molecular and biological functions of lncRNAs are important in providing new insights into the treatment of Dox-induced cardiac injury. The results suggested that MIF pretreatment induced LncRNA–NEAT1 accumulation in exosome^MIF^ derived from MSCs, with a significant anti-senescent effect against Dox-induced cardiac injury. LncRNA–NEAT1 is a 2.1-kb lncRNA transcribed from the *NEAT1* gene [34]. *NEAT1* is important for gene stability [35]. A previous study showed that Dox promoted cellular damage though impairing gene stability [36]. Moreover, silencing lncRNA–NEAT1 in MSCs, even MSCs treated with MIF, could not exert a cardioprotective effect, with shortened telomere length and impaired telomerase activity, both important in gene stability [37].

In recent years, emerging evidence suggested that lncRNAs served mainly as a miRNA sponge to exert their post-transcriptional functions as ceRNAs, which is more effective than the traditional anti-miRNA approach [38]. Accordingly, starBase database prediction and dual-luciferase reporter gene assay suggested that exosome^MIF^ exerted an anti-senescent effect against Dox in vitro by inhibiting miR-221-3p expression, thus influencing the expression of the downstream gene *Sirt2*. Mir-221-3p was notably activated in the age-related degeneration diseases [39]. MiR-221-3p is enriched in aged cardiac tissue, thus leading to age-related cardiac injury [40]. Dox induced cardiac senescence accompanied by elevated levels of miR-221-3p in the cardiac tissue, while this elevation of miR-221-3p was reversed by exosomes^MIF^, through transferring LncRNA–NEAT1. Previous studies proposed that miR-221-3p upregulation during replicative senescence acted in concert to induce cell cycle phase arrest and telomere erosion, establishing a senescent phenotype [24]. In our research, overexpression of miR-221-3p not only re-settled the cell cycle, but also shortened the telomere length and inactivated the telomerase activity, which were recovered by exosomes^MIF^.

In this study, the bioinformatics analysis showed that miR-221-3p interacted with the 3′-UTR of Sirt2 and suppressed Sirt2 expression at the post-transcriptional level, which was confirmed by the results of the luciferase reporter assay. Aging is one of the key risk factors for cardiac injury; the Sirt modulate lifespan in species ranging from yeast to mammals [41, 42]. Sirt are a class of deacetylase enzymes that play an important role in tissue regeneration [43]. Sirt2 has been largely studied in the context of aging and aging-associated diseases [44]. Sirt2 deficiency is known to promote genomic instability, which is an important event in cellular senescence [45]. The present study also found that Sirt2 silencing impaired the anti-senescent effect of exosomes^MIF^. Sirt2 has been suggested as an AMP-Activated Protein Kinases (AMPK) activator, thus alleviating age-related cardiac hypotrophy [46]. Meanwhile, AMPK activation could attenuate Dox-induced cardiotoxicity [47]. A previous study found that MIF could exert a rejuvenation effect through AMPK activation [48]. These findings suggested that Sirt2 took part in exosome^MIF^ against Dox-induced cardiac injury.

Some limitations existed in the current study. First, only LncRNA-NEAT1 in exosome^MIF^ was explored. Further investigation is required to explore the functions of other LncRNAs which are significantly changed in exosome^MIF^. Second, it would be important to confirm exosome^MIF^ dependent biological pathways also in heart tissue of model of tumor. Last but not least, miR-221-3p KO animal will be applied to verify our findings in the future study.

## Conclusions

The present study demonstrated that exosome^MIF^ relieved Dox-induced cardiomyocyte senescence, and these beneficial effects were mediated by the novel exosome/lncRNA–NEAT1/miR-221-3p/Sirt2 pathway. Hence, it proposed a novel ceRNA signaling pathway to optimize the stem cell–based anti-senescent effect. Given that exosomes are easy to obtain, exosome-mediated therapy represents a potentially useful approach for clinical applications in Dox-induced cardiac injury.

## Materials and methods

### Animals

Male C57/Bl6 mice were maintained in accordance with the guidelines published by the US National Institutes of Health. All study procedures were approved by the Institutional Animal Care and Use Committee of Wenzhou Medical University (IRB Approval Number: wydw 2019–0491). This study was conducted in compliance with the Guide for the Care and Use of Laboratory Animals published by the National Academy Press (NIH, revised in 1996).

### Treatment using Dox and exosome^MIF^ in vivo

In eight-week-old male C57/Bl6 mice, Dox was injected in three intraperitoneal (i.p.) injections (4 mg/kg body weight) on alternative days in a time span of 1 week (Monday, Wednesday, and Friday) for a cumulative dose of 12 mg/kg. For exosome^MIF^ treatment groups, exosome^MIF^ were injected (i.p.) on alternative days between Dox treatments (Tuesday, Thursday, and Saturday), as previously reported [32]. After the experiments, mice were sacrificed by CO_2_ inhalation. The investigator was blinded to the group allocation during the experiment.

### Echocardiographic evaluation

For echocardiography, 14 days after treatment with Dox, mice were anesthetized with 1.5–2% isoflurane and kept warm on a heated platform. The vital signs were monitored during echo. The vevo 2100 was used to observe the echocardiographic parameters of mice and assess the cardiac functions (Vevo 2100, Visual Sonics, Canada).

### Quantitative reverse transcription-polymerase chain reaction (qRT-PCR)

RNA was isolated using TRIzol reagent (Invitrogen, CA, USA), and cDNA was synthesized using an ImProm-II reverse transcription kit (Promega, WI, USA) following the manufacturer’s instructions. Quantitative reverse transcription-polymerase chain reaction (qRT-PCR) was performed with SYBR Green to detect mRNA levels. The mRNA levels were calculated relative to the control Gapdh or U6 using the 2^−ΔΔCq^ method. The sequences of all qRT-PCR primers are shown in Table 1.

**Table. 1:** Primer sequences

Genes	Sequences
p27	F: 5′-CCTGGAGCGGATGGACGCCAGACA-3′
	R: 5′-CACCAAATGCCGGTCCTCAGAGTT-3′
p16	F: 5′-TTG GCC CAA GAG CGG GGA CA-3′
	R: 5′-GCG GGC TGA GGC CGG ATT TA-3′
p21	F: 5′-TCCACAGCGATATCCAGACA-3′
	R: 5′-GGACATCACAGGATTGGAC-3′
Sirt1	F: 5′-TTGGCACC GATCCTCGAAC-3′
	R: 5′-CCCAGCTCCAGTCAGAACTAT-3′
Sirt2	F: 5′-TGAATGGCACCTACAGAGAC-3′
	R: 5′-CAAAGGCATTATGGTAGGGC-3′
Sirt6	F: 5′-AGTTCGACACCACCTTTGAG-3′
	R: 5′-CGTACTGCGTCTTACACTTG-3′
LncRNA–NEAT1	F: 5′-TTGGGACAGTGGACGTGTG-3′
	R: 5′-TCAAGTGCCAGCAGACAGCA-3′
miR-221-3p	F: 5′-GGGAAGCTACATTGTCTGC-3′
	R: 5′-CAGTGCGTGTCGTGGAGT-3′
Telomere length	F: 5′-TGAAAGTAGAGGATTGCCACTG-3′
	R: 5′-AGCCAGAACAGGAACGTAGC-3′
GAPDH	F: 5′-GGA GCC AAA AGG GTC ATC AT-3′
	R: 5′-GTG ATG GCA TGG ACT GTG GT-3′
U6	F: 5′-CTCGCTTCGGCAGCACA-3′
	R: 5′-AACGCTTCACGAATTTGCGT-3′
siRNA–LncRNA–NEAT1	5′-TACCATCAGCCTTTAG-3′
siRNA–LncRNA-NT	5′-AACACGTCTATACGC-3′
siRNA–Sirt2	5′-GCAGCUUGUGUGAGCUCAATT-3′
siRNA-NT	5′-UUGAGCUCACACAAGCUGCTT-3′

### Microarray analysis

Exosomes derived from MSCs, or MSCs treated with MIF, were lysed immediately in 500 μL of TRIzol reagent (ThermoFisher Scientific, MA, USA) and stored at − 80 °C before purification using a standard phenol–chloroform extraction protocol with an RNAqueous Micro Kit (ThermoFisher Scientific). The transcriptome was subjected to microarray analysis using an Affymetrix human array (ThermoFisher Scientific) and normalized based on quantiles.

### Western blot analysis

Western Blot analysis was conducted as previously described [49]. Primary antibodies, including p27^kip^ (ab62364), p16^INK4a^ (ab211542), Flotillin-1 (ab41927), CD81 (ab109201), Sirt2 (ab211033), and β-actin (ab179467), were purchased from Abcam.

### Cardiomyocyte isolation and culture

Mouse ventricular myocytes were isolated from C57BL/6 mice. The animals were euthanized by cervical dislocation. The hearts were obtained and digested in a digestion solution containing 0.25% trypsin and collagenase I. Myocytes were separated after 3 h of differential sedimentation and adhesion. They were then cultured in low-glucose Dulbecco’s modified Eagle’s medium (DMEM) containing 10% fetal bovine serum (FBS).

### Senescence-associated β-galactosidase assay (SA-β-gal assay)

The cellular senescence was measured using the SA-β-gal assay (Cell Signaling Technology, MA, USA). Briefly, the cells at the density of 2 × 10^4^ were fixed with 2% paraformaldehyde and incubated with the SA-β-gal staining solution as previously described [50].

### Agomir studies

Chemically modified oligonucleotides were designed to mimic miR-221-3p. Eight-week-old male C57/Bl6 mice were injected (intraperitoneally) with agomir-221-3p (80 mg/kg) or control agomir (80 mg /kg) for three consecutive days as previously reported [51].

### Cell culture and cell treatment

BM‑MSCs were isolated using a standard protocol, as described previously [52]. Briefly, bone marrow was isolated from the femur and tibia of mice by flushing with PBS. Adherent MSCs were propagated and maintained at 37˚C and in the presence of 5% CO_2_ in high-glucose DMEM supplemented with 10% FBS and 1% penicillin/streptomycin.

For MIF treatment, the cells were cultured with a medium containing 100 ng/mL of recombinant MIF and incubated at 37 °C for various periods as previously reported [48].

HL-1 murine cardiomyocytes were maintained in fibronectin-coated flasks, supplemented with 10% FBS, 100 U/mL penicillin, 100 mg/mL streptomycin, and 2 mM L-glutamine, and kept semi-confluent at all times.

### Isolation of exosomes

Exosomes were isolated and purified from the supernatants of MSCs and MIF-treated MSCs. The supernatants were collected after 48-h culture. The exosome quick extraction solution was added to the filtered solution at a 1:5 ratio and stored at 4 °C for at least 12 h. The precipitation (exosomes) was dissolved in PBS and stored at − 70 °C. The characterization of exosomes was carried out as previously reported [19].

### Cell cycle assay

Cold anhydrous ethanol (70%) was employed to fix the cells. Then, the cells were treated with propidium iodide (Sigma, MO, USA) and RNase A. A flow cytometer equipped with CellQuest software was used to detect the cell cycle distribution.

### Relative telomere length measurement

Relative telomere length quantification in HL-1 cells was performed using a qPCR approach based on a previously established method [53], using Gapdh as the normalizing gene. The primer pairs used to detect the telomere length are listed in Table 1.

### Relative telomerase activity measurement

The telomerase activity of HL-1 cells was examined using a Telo TAGGG Telomerase PCR Enzyme-linked immunosorbent assayPlus kit according to the manufacturer’s instructions as described previously [54].

### Flow cytometric analysis of cell apoptosis

Annexin V-FITC Apoptosis Detection Kit was applied to test cellular apoptosis, according to manufacturer’s protocol. Briefly, harvested cells were resuspended in 300 µL binding buffer, and incubated with 5 µL Annexin V-fluorescein isothiocyanate (FITC) solution for 30 min at 4ºC in dark conditions, followed by further incubation in 5 µL propidium iodide for 5 min. The cells were then analyzed immediately by bivariate flow cytometry using a BD FACSCanto II equipped with BD FACSDiva Software (Becton–Dickinson, San Jose, CA, USA).

### Fluorescence tracing of exosomes in vitro

Exosomes were labeled with DiI incubated with the dye (1 mM) at the volume ratio of 500:1 for 30 min, followed by exosome isolation. For the in vitro tracing of exosomes, cardiomyocytes were incubated with DiI-labeled exosomes for 3 h. The cell nuclei were stained with DAPI (1:1000, Invitrogen) for 10 min at 37 °C. Fluorescence was detected under a microscope.

### Small interfering RNA transfection

LncRNA-NEAT1 expression in MSCs was knocked down using small interfering (si)RNAs, with a nontargeting siRNA as a negative control (Invitrogen). Sirt2 expression in cardiomyocytes was also knocked down using siRNAs. The procedures were conducted as described previously [54]. The transfection efficiency was detected using qRT-PCR and western blot analysis.

### MiR-221-3p overexpression

Cardiomyocytes were seeded into six-well plates at a density of 1 × 10^5^ cells per well. The cells were transfected with miR-221-3p mimic or negative control (NC) mimic (Pre-miR miRNA Precursors, Life Technologies, Karlsruhe, Germany) using X-treme transfection reagent (Roche Applied Science, Penzberg, Germany), according to the manufacturer’s protocol, to induce the overexpression of miR-221-3p. The cells were harvested for further analysis 48 h after transfection, and the transfection efficiency was analyzed using qRT-PCR.

### Pulldown assay with biotinylated miRNA

Cardiomyocytes were transfected with biotinylated miRNA, collected 48 h after transfection. The cell lysates were incubated with M-280 streptaviden magnetic beads (Invitrogen, San Diego, CA, USA) as described previously [55, 56]. RT–qPCR was applied to analyze the bound RNAs, purified using TRIzol reagent (Invitrogen).

### Luciferase reporter assay

The 3′-untranslated regions (UTRs) of LncRNA–NEAT1 and Sirt2 were synthesized, annealed, and inserted into the *Sac*I and *Hind*III sites of the pmiR-reporter luciferase vector (Ambion), downstream of the luciferase stop codon to induce the mutagenesis of LncRNA–NEAT1 and Sirt2. The constructs were validated by sequencing. Cardiomyocytes were seeded into a 24-well plate for luciferase assay. After overnight culture, the cells were co-transfected with a wild-type (WT) or mutant plasmid and an equal amount of miR-221-3p mimic or miR-NC mimic. Luciferase assays were performed using a dual-luciferase reporter assay system (Promega) 24 h after transfection.

### Statistical analysis

Data were expressed as the mean ± standard deviation (SD). Differences between groups were tested by one-way analysis of variance, and comparisons between the two groups were evaluated using the Student’s *t* test. Analyses were performed using SPSS package v19.0 (SPSS Inc., IL, USA). A *P* value less than 0.05 was considered statistically significant.

## Supplementary information

**Additional file 1: Figure. S1.** Confirmation of exosomal collection. The exosome was characterized by TEM (A), NTA (B), and western blot (C).

**Additional file 2: Figure S2.** MiR-221-3p directly targeted Sirt2. (A) Binding sites of miR-221-3p and the Sirt2 3′-UTR. (B) Dual-luciferase reporter was applied in cardiomyocytes after co-transfection with miR-221-3p mimic, miR-NC mimic, and Sirt2 3′-UTR wild-type (WT) or mutant (MUT) plasmids. *P < 0.05 versus the miR-221-3p mimic in the WT group in repeated measures ANOVA, n = 3. (C and D) Sirt2 and β-actin protein levels were confirmed using western blot analysis in cardiomyocytes. Untreated cardiomyocytes were used as control. *P < 0.05 versus Control; ^▲^*P* < 0.05 versus Dox; ^○^*P* < 0.05 versus Dox + exosome^MIF^ + miR-221-3p mimic in repeated measures ANOVA, n = 3.

## Data Availability

All data and materials are available in the manuscript.

## Abbreviations

AMPK: AMP-activated protein kinases
ceRNAs: Competing endogenous RNAs
DIC: Dox-induced cardiomyopathy
DMEM: Dulbecco’s modified Eagle’s medium
Dox: Doxorubicin
EF: Ejection fraction
FBS: Fetal bovine serum
FACS: Flow cytometric analysis cell scan
FS: Fractional shortening
lncRNAs: Long non-coding RNAs
MIF: Macrophage migration inhibitory factor
MSCs: Mesenchymal stem cells
miR: microRNA
NEAT1: Nuclear paraspeckle assembly transcript 1
qRT-PCR: Quantitative reverse transcription-polymerase chain reaction
ROS: REACTIVE oxygen species
SA-β-gal assay: Senescence-associated β-galactosidase assay
SD: Standard deviation
siRNA: Small interfering RNA
Sirt2: Sirtuin 2
3′-UTRs: 3′-Untranslated regions
