# Supplementary figures and images for: Exosomal LncRNA–NEAT1 derived from MIF-treated mesenchymal stem cells protected against doxorubicin-induced cardiac senescence through sponging miR-221-3p
Source: J Nanobiotechnology. 2020 Oct 31;18:157. doi: 10.1186/s12951-020-00716-0 (PMC7603694; doi:10.1186/s12951-020-00716-0)

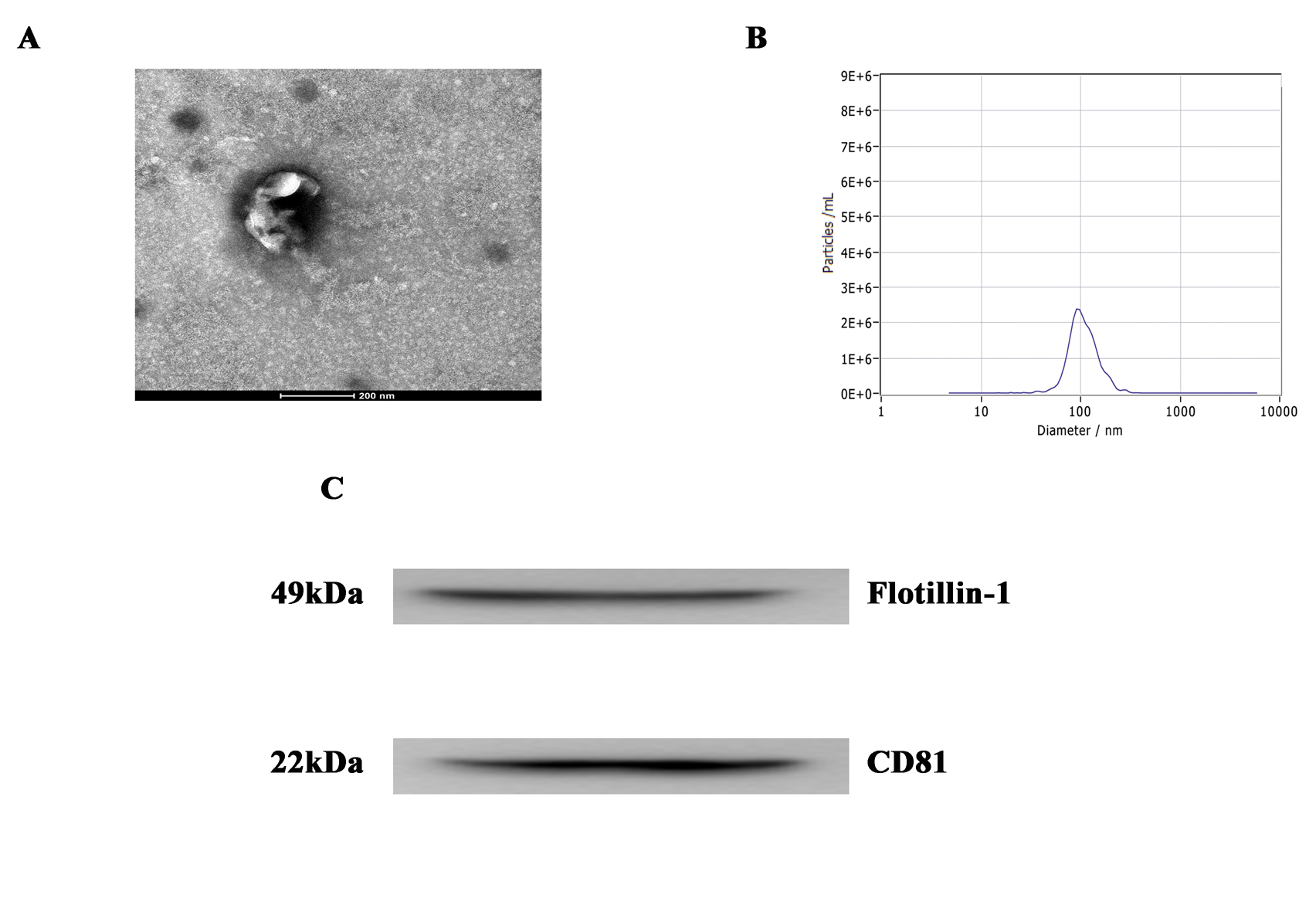

Supplement: Supplementary file 1 — Additional file 1: Figure. S1. Confirmation of exosomal collection. The exosome was characterized by TEM (A), NTA (B), and western blot (C). [file 12951_2020_716_MOESM1_ESM.tif]

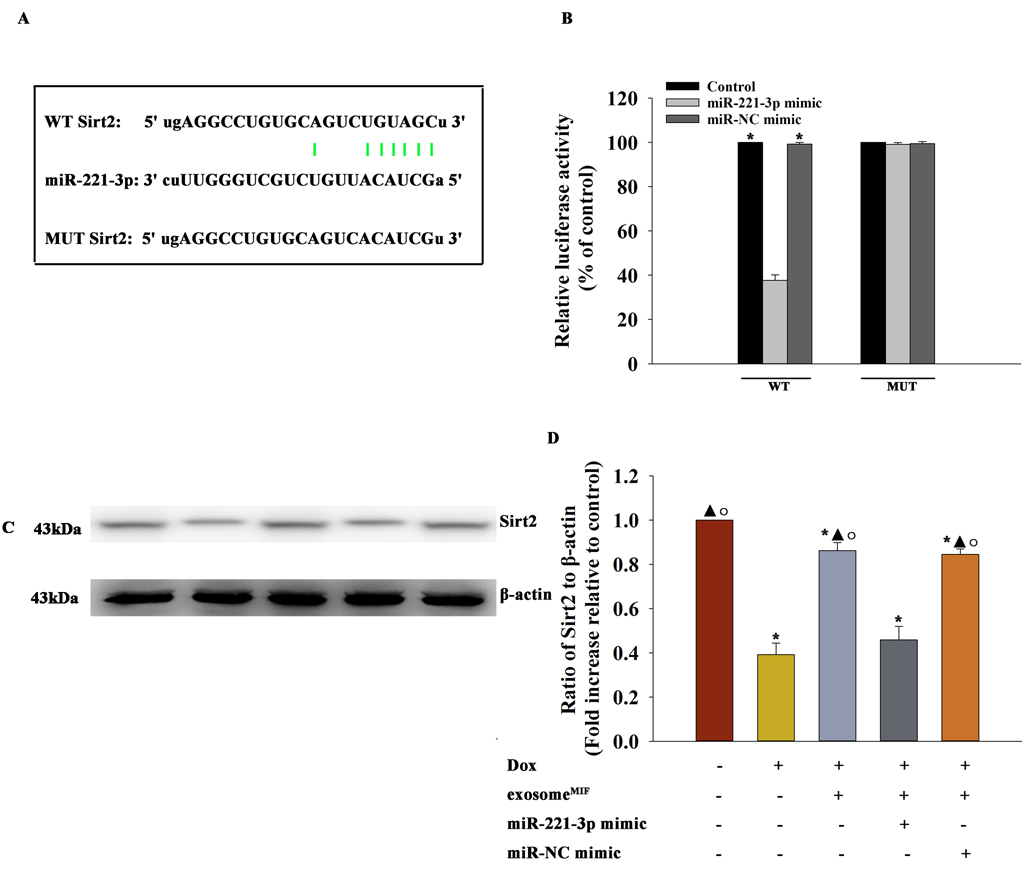

Supplement: Supplementary file 2 — Additional file 2: Figure S2. MiR-221-3p directly targeted Sirt2. (A) Binding sites of miR-221-3p and the Sirt2 3′-UTR. (B) Dual-luciferase reporter was applied in cardiomyocytes after co-transfection with miR-221-3p mimic, miR-NC mimic, and Sirt2 3′-UTR wild-type (WT) or mutant (MUT) plasmids. *P < 0.05 versus the miR-221-3p mimic in the WT group in repeated measures ANOVA, n = 3. (C and D) Sirt2 and β-actin protein levels were confirmed using western blot analysis in cardiomyocytes. Untreated cardiomyocytes were used as control. *P < 0.05 versus Control; ▲P < 0.05 versus Dox; ○P < 0.05 versus Dox + exosomeMIF + miR-221-3p mimic in repeated measures ANOVA, n = 3. [file 12951_2020_716_MOESM2_ESM.tif]
